# A two-step bioconjugation of *S. aureus* lipoteichoic acid (LTA) affords fluorescent probes that illuminate the interaction between Gram-positive glycolipids and mammalian cell membranes

**DOI:** 10.1039/d6cb00113k

**Published:** 2026-05-26

**Authors:** Jake Henry, Joe Nabarro, Harriet Chidwick, Georgia Conrich-Wilks, Emily G. Morton-West, Alice Crook, Dave Boucher, Nathalie Signoret, Martin A. Fascione

**Affiliations:** a Department of Chemistry, University of York York UK martin.fascione@york.ac.uk; b Department of Biology University of York, Heslington York YO10 5DD UK; c Experimental Medicine and Biomedicine Group, Hull York Medical School, University of York YO10 5DD UK nathalie.signoret@york.ac.uk; d York Biomedical Research Institute, University of York, Heslington York YO10 5DD UK

## Abstract

Lipoteichoic acid (LTA), a major constituent of Gram-positive bacteria cell wall, is an amphiphilic glycolipid and well-established stimulator of immune cells through activation of Toll-Like Receptor 2 (TLR2) complexes. LTA binding to TLR2 is essential for this process but not the only step required, meaning that new tools are needed to visualize and track LTA interactions with host cells. Here we present a simple aldehyde-based bioconjugation approach to label native LTA purified from *S. aureus*, generating fluorescent LTA derivatives with minimal functional impairment by targeting modification distal to the diacylglycerol lipid anchor that is essential for host cell interactions. We demonstrate that this approach not only facilitates the study of fluorescent LTA binding to established host TLRs, but also reveals an underappreciated propensity of LTA lipids to interact with mammalian membranes independently of TLRs.

## Abstract 2

Oxime-based bioconjugation enables fluorescent labelling of native bacterial glycolipid lipoteichoic acid (LTA), facilitating study of its interaction with mammalian membranes.

## Graphical abstract

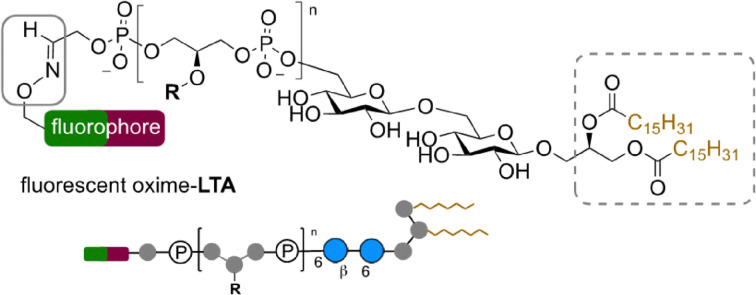

## Introduction

Lipoteichoic acid (LTA) is an amphiphilic glycolipid and pathogen associated molecular pattern (PAMP) presented on the surface of Gram-positive bacteria through membrane anchoring of its diacylglycerol lipids. Currently, there are five identified types of LTA, with Type 1 LTA from *S. aureus* being the best characterized.^1^ Whilst, the exact roles of LTA in bacteria remain incompletely elucidated, it is well established that LTA is essential for bacterial growth and survival, as mutants lacking LTA become very vulnerable to changes to their environment and are more susceptible to antibiotics.^1–3^ With regards to human health, LTA is considered as a virulence factor triggering inflammatory responses and a molecular driver of sepsis, a life threatening condition with more than 48 million cases across the globe, of which 43% are attributable to Gram-positive bacterial infections.^4,5^ LTA mediates bacterial adherence to host tissues for colonisation, but once released from bacteria it activates host defence processes. Mechanistically, stimulation of neutrophils, monocytes and macrophages occurs through LTA loading onto Toll-Like Receptor 2 (TLR2) complexes, to initiate the host response. Importantly accessory host molecules bind and aid in the delivery of LTA to TLR2,^6^ while TLR2-independent effects of LTA have also been reported on immune cells.^7^ Therefore to better understand how LTA exerts its effects on host mammalian cells, reliable tools enabling detection, tracking and functional analysis of this microbial component are required.

**Fig. 1:**
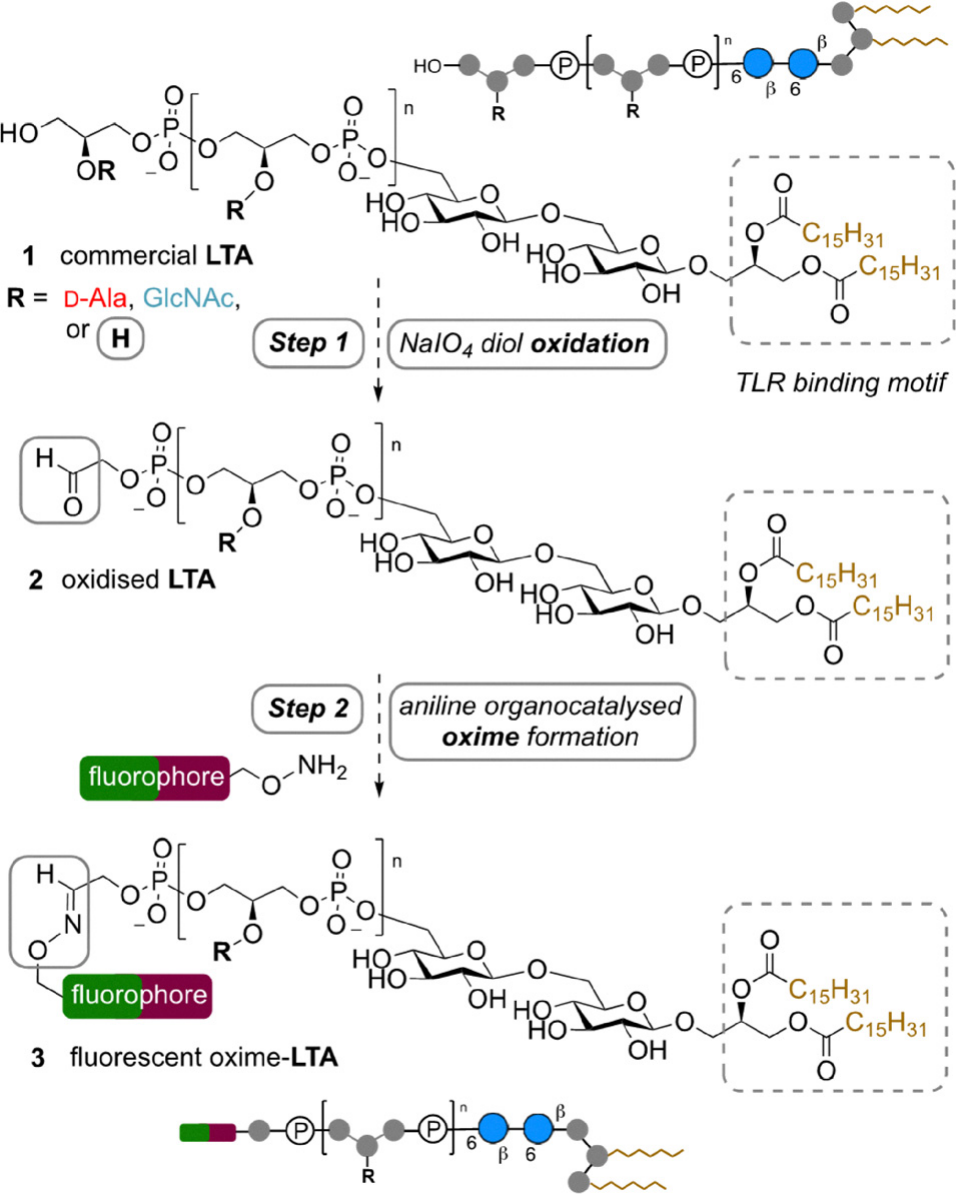
Proposed two-step method for bioconjugation of commercial *S. aureus* lipoteichoic acid (LTA) 1*via* aldehyde 2 to afford fluorescent oxime bioconjugates 3, using a Malaprade oxidation followed by oxime ligation using fluorescent aminoxy reagents.

Fluorescent labelling of biomolecules is an often used chemical biology approach for this application, with electrophilic activated esters and isothiocyanates previously used to label nucleophilic sites on native LTA.^8–13^ However, these approaches can lack region- and site-selectivity, with potentially deleterious effects on the functional activity of the biomolecule. Whilst elegant fully synthetic approaches to LTA have resolved these selectivity issues^14,15^ and delineated the contribution of component parts to biological activity,^16–18^ the formidable multistep synthesis required^19^ can often limit the length of LTA accessible and these approaches to only a few specialised labs. Therefore, herein we bridge these two approaches and use mild and selective aldehyde chemistry to label readily accessible native LTA purified from *S. aureus*1, with the aim of generating fluorescent LTA derivatives with minimal functional impairment by targeting modification distal to the diacylglycerol lipid anchor, which is the key mediator of interaction of LTA host cell receptors, including TLR2, CD14 and CD36.^20,21^ Specifically we describe the application of a simple two-step native LTA bioconjugation protocol consisting of Malaprade periodate vicinal diol oxidation to an aldehyde 2 followed by aniline organocatalysed oxime ligation^22^ of the resulting aldehyde using Alexa Fluor 647 or Alexa Fluor 488 nucleophiles to afford fluorescent bioconjugates 3 (Fig. 1). We demonstrate that as well as being operationally simple enough for labs lacking synthetic chemistry expertise, our approach not only facilitates the study of fluorescent LTA binding to established host TLRs, but also reveals an underappreciated propensity of LTA lipids to interact with mammalian membranes independently of TLRs, with potential ramifications for our understanding of the role of bacterial glycolipids in human infection.

## Results and discussion

**Fig. 2:**
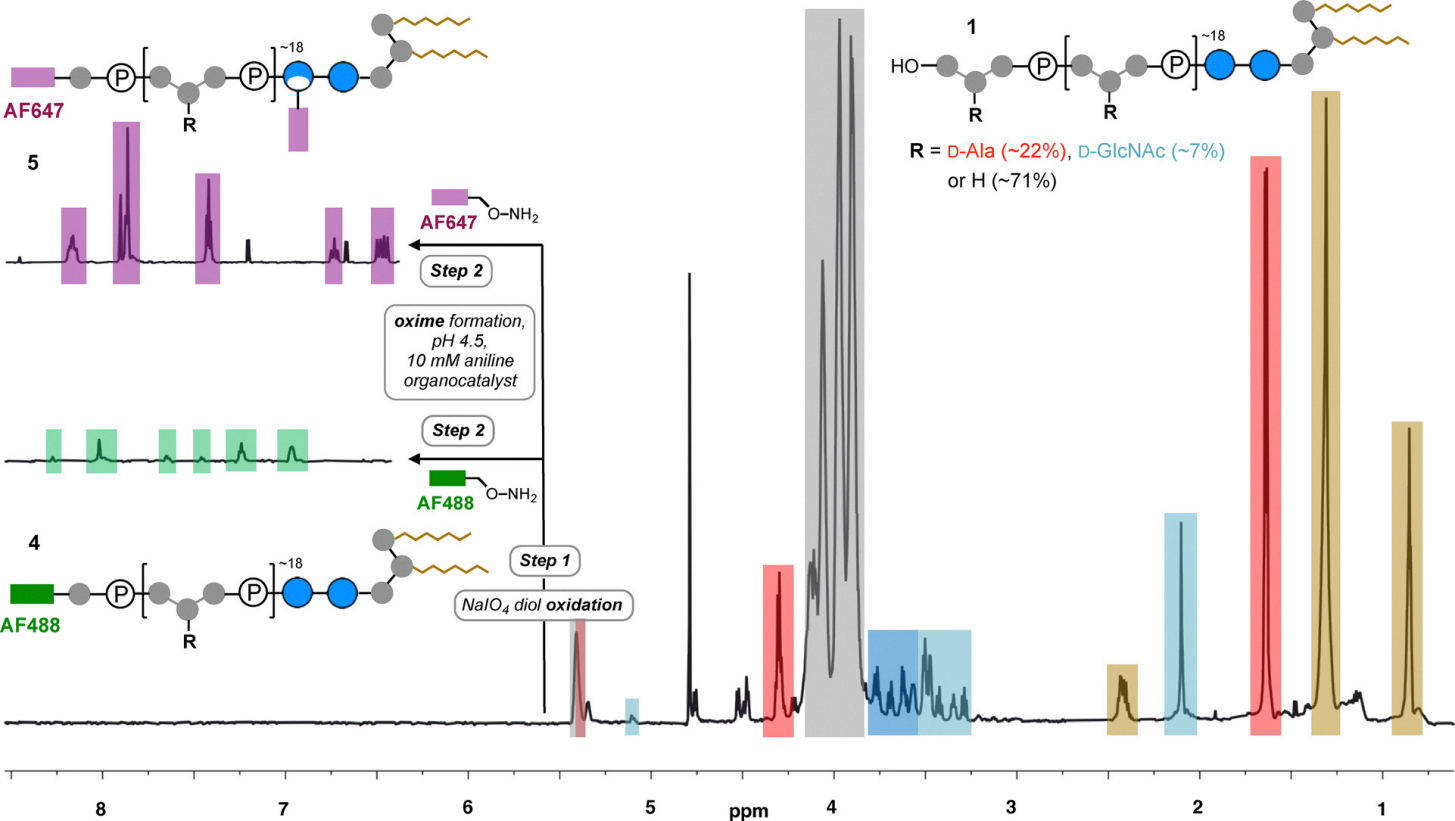
Comparative 700 MHZ DOSY ^1^H-NMR analysis of unmodified LTA 1 (main) and fluorescently modified LTA 4 and 5 (inset, only aromatic region for clarity), highlighting signals for GroP backbone (grey), lipids (brown), gentiobiose core (blue), d-GlcNAc (cyan), d-Ala (red), AF488 (green) and AF647 (purple). Note ‘half-moon’ glucose symbol depiction in 5 denotes a putative ring-opened acyclic dialdehyde species resulting from periodate mediated glucose ring opening.

**Fig. 3:**
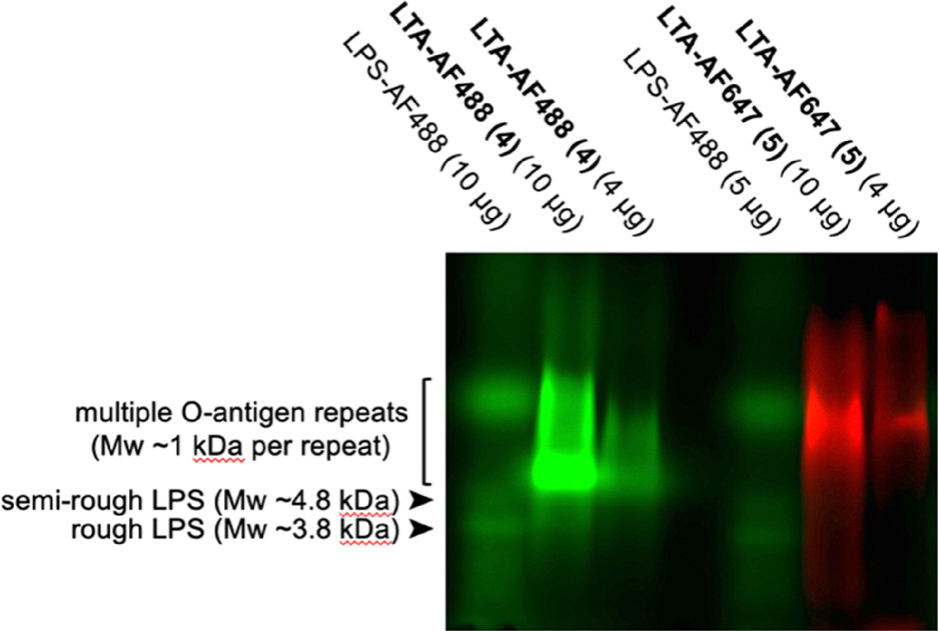
TSDS-PAGE analysis of fluorescent-LTA 4 and 5 compared to an AF-488 labelled lipopolysaccharide (LPS) standard from *E. coli* O111:B4. Composite image analysis following direct in gel detection of two different fluorescent signals.

*S. aureus* LTA 1 is a negatively charged glycolipid consisting of a lipid anchor bearing two fatty acid chains (Fig. 1) attached to a gentiobiose disaccharide core (Glc(β1-6)Glc) that is modified at the O6 with a backbone of glycerol phosphate (GroP) repeating units which can be substituted with either d-GlcNAc glycoside, or d-Ala esters. Both fatty acids are essential for TLR binding and subsequent release of immunostimulatory cytokines, with the length of the backbone^17^ and d-Ala substitution also contributing to immunostimulatory potency of LTA.^18,23^ Although LTA was first purified from Gram-positive bacteria using hot phenol–water extraction for biological studies,^24^ its activity as a bacterial ‘endotoxin’ remained controversial for decades due to the presence of contaminants, and the subsequently proven propensity for chemical degradation during purification. Milder extraction with *n*-butanol revealed that this hot phenol degradation often results in the reduction of the GroP backbone length and d-Ala substitution and loss of immunostimulatory activity.^23^ LTA can be synthesised, however this requires specialist laboratory equipment and chemistry expertise, making it inaccessible to many laboratories.^14^ Therefore, commercially available active LTA extracted from *S. aureus* with *n*-butanol, as available from Invivogen, has thus become the gold standard reagent for biological study of LTA signalling. We therefore opted to use this supply as a starting point for chemical bioconjugation of the LTA backbone. Initial 700 MHz diffusion-ordered spectroscopy (DOSY) ^1^H-NMR analysis of the commercial *S. aureus* LTA in our hands revealed an average length of ∼18 GroP repeats, using the average number of protons in the lipid anchor as a reference,^25^ (with ∼22% d-Ala ester substitution and ∼7% d-GlcNAc glycoside substitution of the GroP backbone (Fig. 2). Interestingly DOSY NMR, which separates NMR peaks based on their diffusion coefficient and therefore molecular weight^26^ revealed contamination of the commercial sample with faster diffusing small d-Ala not attached to the slower diffusing larger molecular weight LTA, suggesting some cleavage of the d-Ala ester on the backbone (loss of ∼5% substitution, SI Fig. S1) had occurred during purification or handling, as also occurs at pH 8.5 or above.^18^ Considering the calculated level of GroP substitution we determined on average that ∼71% of the heterogenous samples backbone would be unsubstituted (R = H). Therefore, on average the majority of the terminal LTA GroP unit in this sample could be a considered as a vicinal diol bearing a primary alcohol, a functionality potentially susceptible to relatively rapid Malaprade periodate oxidation (Fig. 1, step 1) compared to other diols in the glycolipid.^27,28^ Mild periodate oxidation of this terminal diol would afford oxidised LTA 2 bearing an electrophilic aldehyde moiety which could then be derivatised *via* oxime ligation using reactive α-effect nucleophiles under mild conditions, using aniline as an accelerating water soluble organocatalyst.^29^ To realise this bioconjugation, we therefore subjected LTA 1 (2 mg mL^−1^) to 5 mM NaIO_4_ in 200 mM NaOAc buffer at pH 5.5 for two hours on ice, before dialysis into pH 4.5 buffer and incubation for two hours following addition of 10 mM aniline and either hydroxylamine-Alexa Flour 488 or hydroxylamine-Alexa Flour 647 fluorophores, in a fivefold excess over LTA. Following further dialysis into water and lyophilisation, the resultant 488 and 647 fluorescent oxime-LTA bioconjugates 4 and 5 were then characterised by 700 MHz DOSY ^1^H-NMR to determine the efficiency of labelling (Fig. 2, inset). ^1^H-NMR signals associated with the fluorophores in the bioconjugates were easily identifiable in the aromatic region through comparison to the unconjugated starting materials (SI Fig. S2 and S3), with the DOSY NMR experiments also confirming that the fluorophores in the sample diffused similarly to the higher molecular weight LTA, indicative of covalently attachment. Using the lipid anchor as a reference again, the labelling efficiency for AF488-LTA 4 was calculated as ∼75% fluorophore per LTA molecule, approximately consistent with the previously determined ∼71% unsubstituted GroP backbone. Although the heterogeneity of the starting material precludes unequivocal characterisation of the site of fluorophore attachment, considering the rate of Malaprade oxidation is governed by the steric accessibility, type and stereochemical configuration of the reactive diols, we would expect the terminal vicinal diol of LTA to be the primary site of oxidation and also the least sterically hindered for subsequent oxime ligation, and thus the likely site of mono-modification in AF488-LTA 4. However, NMR analysis revealed AF647-LTA 5 is labelled with ∼187% fluorophore per LTA molecule, indicating at least another site of attachment, with the only other diols available for Malaprade oxidation in the glucose containing gentiobiose (Glc(β1-6) disaccharide core (discounting the low level of ∼7% GlcNAc glycoside GroP substitution). Although the *trans* orientation of C2–C3 and C3–C4 diols in glucose are among the slowest sites for oxidation among carbohydrates,^30,31^ due to the unfavourable distortion of the glucose ^4^C_1_ chair conformation required for formation of the planar cyclic ester intermediate in the Malaprade reaction, oxidation of glucose to di-aldehydes can occur in the presence of periodate with a rate constant of ∼4 × 10^−2^ mol^−1^ s^−1^.^32^ Therefore, the likely second site of fluorophore attachment in AF647-LTA 5 is within this gentiobiose core, but notably both the terminal diol and this disaccharide core are distal to the lipid anchor which mediates cell surface TLR interactions. To further characterise bioconjugates 4 and 5 we subsequently performed polyacrylamide gel electrophoresis (PAGE) analysis using Tricine-SDS PAGE gel (Fig. 3), including AF488 labelled lipopolysaccharide from *E. coli* O111:B4 as a ladder^33^ to aid analysis of the size of our modified LTA using fluorescent visualisation. Notably we observed a smeared banding with our fluorescent LTA consistent with previous LTA analysis,^34^ and a migration through the gel for AF488-LTA 4 consistent with a molecular weight of ∼5–6 kDa. This molecular weight is consistent with the ∼4.8 kDa calculated based on the average LTA length (∼18 GroP repeats) determined by NMR analysis (Fig. 2). Although AF647-LTA 5 appears to migrate more slowly, this could be considered a consequence of that attachment of the additional negatively charged (AF647) fluorophore.

Although combined ^1^H-NMR and PAGE analyses of the modifed LTA indicated bioconjugation had successfully resulted in fluorophore attachment whilst maintaining the structural integrity of the glycolipid, it remained to assess the functional activity of the fluorescent LTA bioconjugates in mammalian cells stimulation. This activation is governed by initial binding to cell surface TLR2 complexes (TLR2/6 or TLR1/2), which then triggers intracellular signalling pathways leading to the secretion of proinflammatory cytokines.^35–37^

**Fig. 4:**
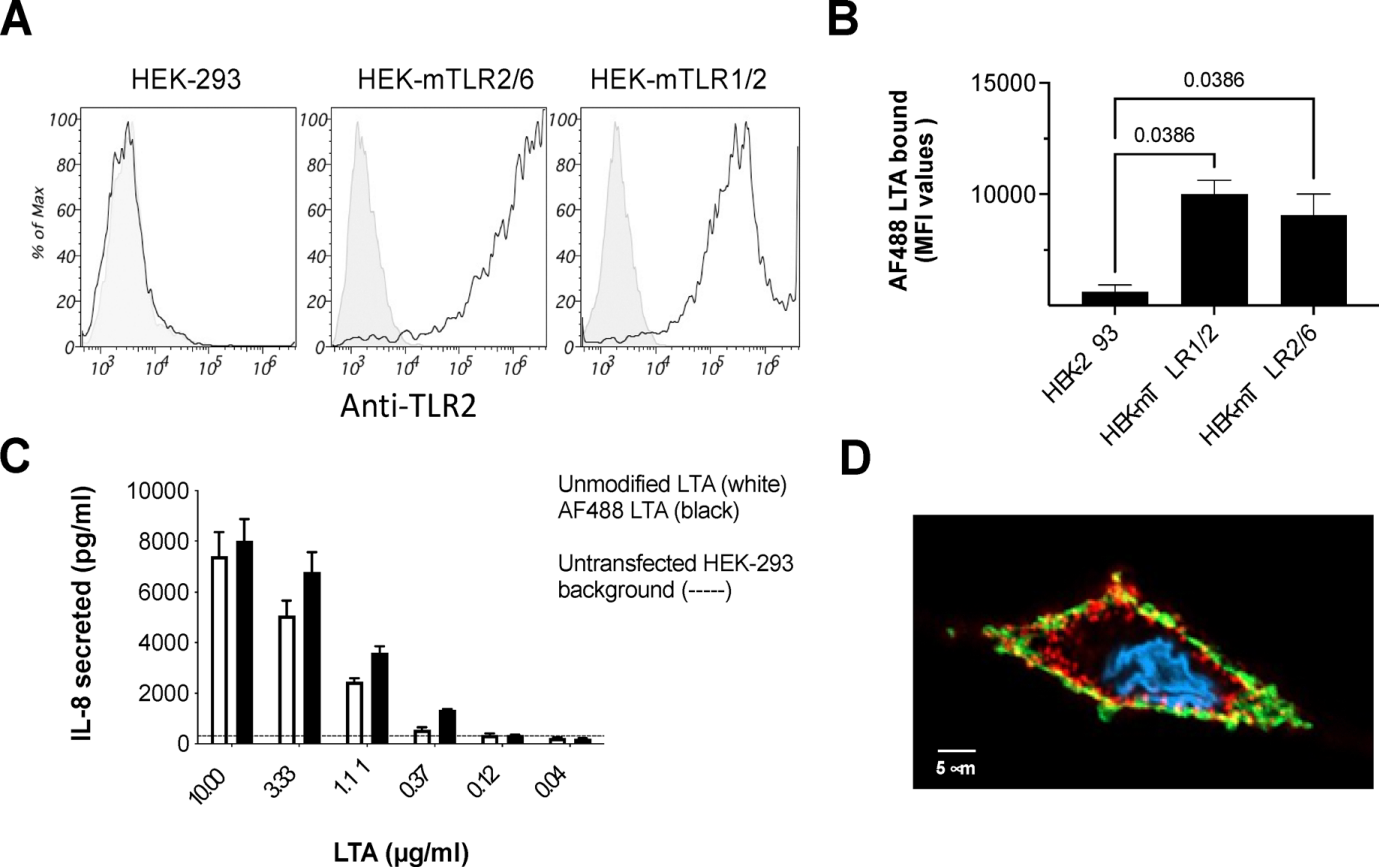
Biological characterization of AF488-LTA compared to unmodified LTA. (A) Flow cytometry detection of TLR2 on HEK-293 control and reporter cells expressing either of the functional mouse TLR2 receptor pairs. Gray peak represents cells stained with secondary antibody only, while white peak represents cells labelled with anti-TLR2 antibody prior to secondary antibody staining. (B) Binding of AF488-LTA (10 µg mL^−1^) following a 90 min cell incubation at 4 °C; The graph reports the Mean Fluorescent Intensity (MFI) signal of cell-bound AF488-LTA, with mean ± sd from two independent triplicate experiments, one way ANOVA statistical analysis and adjusted *q* values. This experiment was performed using a Cytoflex S flow cytometer. (C) Comparative analysis of dose-dependent TLR-2 mediated IL-8 secretion by unmodified and AF488-LTA on HEK-mTLR2/6; IL-8 ELISA performed at highest LTA dose on HEK-293 cells set background level (dotted line). (D) Single confocal section images of an MDM exposed to 10 µg mL^−1^ AF488-LTA (green) for 2 h at 4 °C and stained for surface TLR2 (red) with DAPI nuclear stain (blue)- scale bar = 5 µm.

To validate the biological activity of the bioconjugates we therefore used HEK-293 reporter cell lines expressing murine TLR1/TLR2 or TLR2/TLR6 pairs, which secrete the cytokine IL-8 upon TLR activation, with non expressing HEK-293 LacZ (HEK-293) cells for negative control.^38^ Flow cytometry analysis initially confirmed the high TLR-2 expression on reporter cells using an anti-TLR2 antibody (Fig. 4A), and following treatment with 10 µg mL^−1^ AF488-LTA 4 also demonstrated binding of the fluorescent green LTA to TLR2/6 and TLR1/2 expressing cells, evidenced by the significant increase in AF488 mean intensity fluorescence (MFI) values compared to non-expressing HEK-293 cells (Fig. 4B). To further demonstrate the functionality of AF488-LTA 4 we tested its ability to induce TLR2 dependent cytokine secretion compared to unmodified LTA 1 (Fig. 4C). HEK-293 expressing the mTLR2/6 pair were stimulated with increasing dose of LTA 1 or AF488-LTA 4 in medium at 37 °C for 24 hours before measuring IL-8 production by performing an ELISA on collected cell supernatants.^38^ The ELISA revealed a similar dose response for AF488-LTA 4 and unmodified LTA 1, with minimal signal for control HEK-293 cells with either form of LTA (background line on graph), indicating the bioconjugation had not impaired the functional activity of the LTA. Additionally, we tested the ability of AF488-LTA 4 to bind TLR2 on cells endogenously expressing receptor complexes by seeding human monocyte-derived macrophages (MDMs) on coverslips before staining for TLR2 surface localisation, exposure to 10 µg mL^−1^ AF488-LTA 4 and analysis by confocal microscopy (Fig. 4D).

Notably, although we observed cell surface coincidence (yellow) of AF488-LTA (green) and TLR2 staining (red), we also observed cell surface binding of AF488-LTA 4 (green) at the membrane where no TLR2 was detected, suggestive of a TLR2-independent interaction of the glycolipid with mammalian cells.

**Fig. 5:**
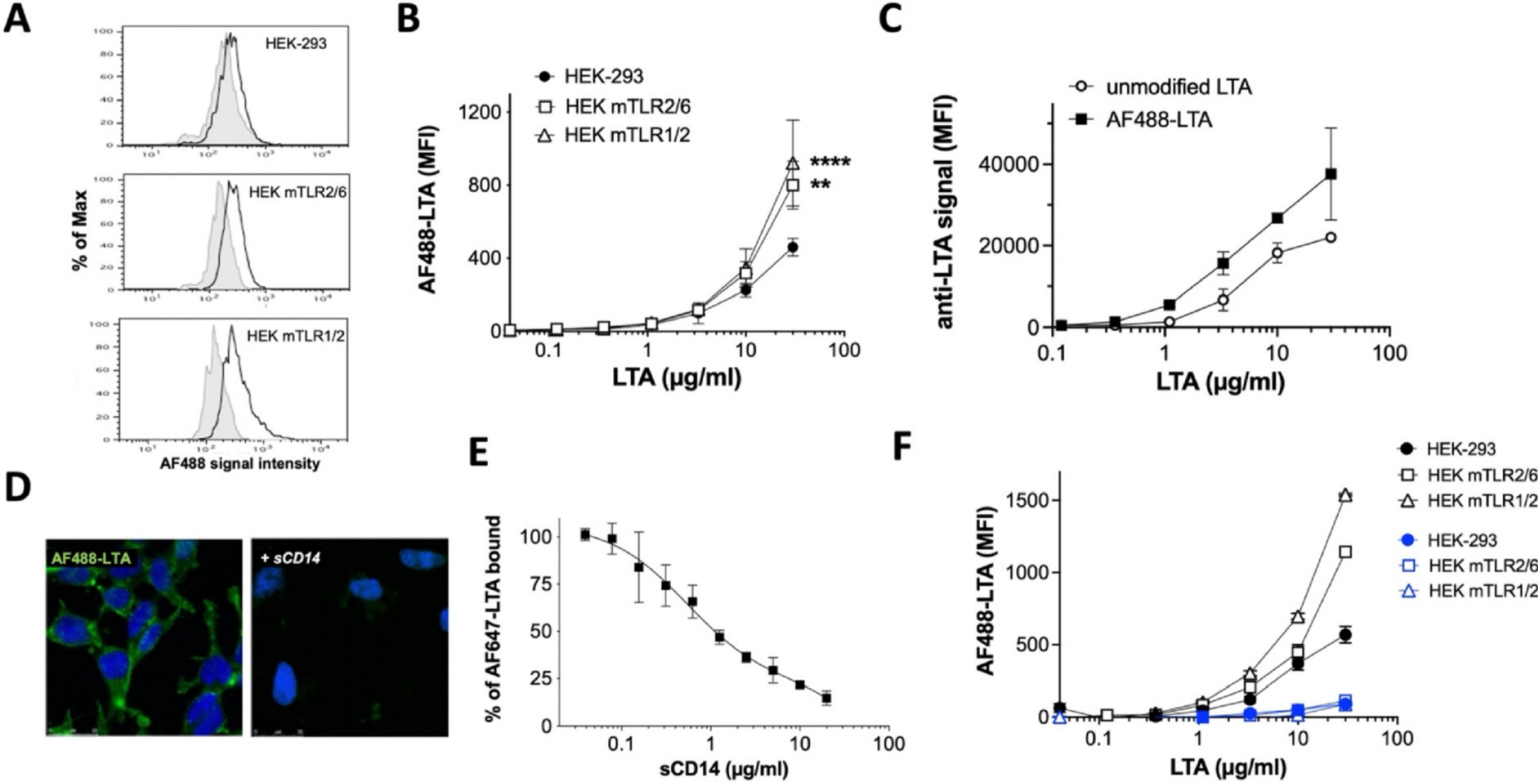
TLR2-dependent and -independent cells interactions. (A) Flow cytometry histogram overlays of HEK-293, HEK-mTLR-2/6 or HEK-mTLR1/2 cells unlabelled (grey filled traces) or exposed to 3.3 µg mL^−1^ AF488-LTA (black open traces) for 90 minutes on ice, showing a more pronounced shift in AF488 signal intensity for TLR2 expressing cells. (B) comparative binding titration of AF488-LTA on the different cell lines, triplicate experiment with two way ANOVA mean comparison to HEK-293 control plus Dunnett's secondary test, **** < 0.0001 ** < 0.005. (C) Comparative binding titration of unmodified LTA and AF488-LTA by indirect fluorescent detection using an anti-LTA antibody. (D) Epifluorescence images with DAPI Fluorescent DNA stain (blue) from HEK-293 cells treated for 2h at room temperature with 5 µg mL^−1^ of AF488-LTA revealing cell membrane staining (green) not seen when AF488-LTA is pre-exposed to 10 µg mL^−1^ sCD14 (+ sCD14); scale bars = 25 µm. (E) sCD14 dose-dependent inhibition of AF647-LTA binding to HEK-293 cells, results expressed as percent of LTA binding with no sCD14 pre-treatment for a representatitve triplicate experiment. (F) comparative cells binding titration of intact (black lines) and hydrolysed (blue lines) AF488-LTA; graph reports mean ± sd values from a triplicate experiment. All flow cytometry data presented in this figure were acquired on a LSRFortessa X20 instrument.

To explore this potential TLR2-independent mechanism, flow cytometry experiments were revisited using a titration of AF488-LTA 4 on HEK-mTLR2/6 or HEK-mTLR1/2 to HEK-293 control cells (Fig. 5A) to dissect dose-dependent binding. Supporting TLR2-independent binding of LTA, titration experiments using the green fluorescent probe consistently showed a lower but dose-dependent increase in cell-associated fluorescent signal on HEK-293 control cells lacking TLR2, compared to TLR2 expressing cells (Fig. 5B). Furthermore a comparable dose-dependant increase in binding to HEK-293 control cells was also observed using AF647-LTA 5 demonstrating the binding was also fluorophore independent (SI Fig. S4) and also not impacted by modification of the gentiobiose core. Whilst an anti-LTA monoclonal antibody, recognising the glycerophosphate backbone of LTA,^39^ also detected HEK-293 cell-surface associated unmodified LTA 1, as well as AF488-LTA 4 (Fig. 5C). We also used the AF488-LTA 4 to probe interaction with control HEK-293 cells by confocal microscopy, demonstrating that fluorescent LTA clearly interacts with the plasma membrane in the absence of TLR2 (Fig. 5D). An LTA mediated TLR-independent paralysis of T-cells was previously reported with observation of membrane-LTA interactions, which were speculatively attributed to an unknown receptor.^40^ However classical early studies, prior to the discovery of TLRs, suggested that LTA was able to reversibly insert into erythrocyte membranes by virtue of its lipid anchor,^41,42^ a conclusion supported by the more recent demonstration that Gram-negative lipopolysaccharide (LPS) glycolipids can spontaneously insert into model membranes.^43,44^ Therefore to dissect if interaction of our AF488-LTA 4 with the plasma membrane was mediated by the lipid anchor we pre-treated probe 4 with a soluble form of CD14 (sCD14), a host binding partner for LTA,^45^ which like TLR2 is known to engage through binding to the lipid anchor.^46,47^ We observed that sCD14 pre-treatment prevented AF488-LTA interaction with HEK-293 cell-surface by microscopy (Fig. 5D), and had a dose-dependant inhibitory effect on AF647-LTA cell binding by flow cytometry (Fig. 5E). Furthermore, we assessed the impact of hydrolysis of the ester linked lipid chains of both AF488-LTA 4 and AF647-LTA 5, and unequivocally demonstrated abrogation of TLR2-dependant and TLR2-independent binding following lipid cleavage (Fig. 5F and SI Fig. S5), reinforcing the hypothesis that LTA is likely able to interact and insert into mammalian cell membranes in a receptor-independent thermodynamically driven process.

## Conclusion

In this study we have leveraged an understanding of the Malaprade oxidation of glycoconjugates to develop a simple bioconjugation of native LTA that can be completed within a few hours by a non-specialist, enabling us to fluorescently tag the glycolipid using oxime chemistry and commercially available hydroxylamine probes. We demonstrated that this mild two-step bioconjugation had a minimal effect on the structure of the native LTA and no significant effect on biological activity of LTA, with probes still able to bind TLR2 receptors on host cells, and activate receptors to trigger cytokine release. Although some reduction in substitution of the GroP backbone and changes to the gentiobiose core occurred during bioconjugations (SI Fig. S2 and S3), this structural modulation did not impact on the ability of the fluorescent LTA to induce cytokine release, compared to unmodified LTA, which is unsurprising considering previous structure–function studies using synthetic LTA derivatives deemed the gentiobiose core to be unnecessary for immunostimulation.^16^ Furthermore the new fluorescent LTA bioconjugates also facilitated the observation of TLR2-independent binding to the plasma membrane. This predilection of LTA was shown to be dependant on its lipid anchor, suggestive of a membrane insertion mechanism, an underappreciated property of amphiphilic PAMPs.^48–50^ Notably, the biological significance of this process for the host remains to be established and can be now be dissected in future studies using this bioconjugation approach to expand the LTA chemical biology toolkit.

## Author contributions

Jake Henry: investigation, methodology, writing – original draft, writing – review and editing. Joe Nabarro: Investigation, methodology writing – review and editing. Harriet Chidwick: investigation, methodology. Georgina Wilks: investigation, methodology Emily Morton-West: investigation, methodology A Crook: investigation, methodology. Dave Boucher: funding acquisition, supervision, conceptualization, writing – review and editing. Nathalie Signoret: funding acquisition, supervision, conceptualization, visualization, formal analysis, writing – original draft, writing – review and editing. Martin A. Fascione: funding acquisition, supervision, conceptualization, visualization, formal analysis, writing – original draft, writing – review and editing.

## Conflicts of interest

The authors declare no competing financial interest.

## Supplementary Material

CB-007-D6CB00113K-s001

## Data Availability

The data supporting this article have been included as part of the supplementary information (SI). Supplementary information is available. See DOI: https://doi.org/10.1039/d6cb00113k.

## References

[cit1] Percy M. G., Gründling A. (2014). Annu. Rev. Microbiol..

[cit2] Alcántara C., Crespo A., Solís C. L. S., Devesa V., Vélez D., Monedero V., Zúñiga M. (2020). Benefic. Microbes.

[cit3] Oku Y., Kurokawa K., Matsuo M., Yamada S., Lee B.-L., Sekimizu K. (2009). J. Bacteriol..

[cit4] Fleischmann-Struzek C., Rudd K. (2023). Med. Klin., Intensivmed. Notfallmed..

[cit5] Dyck B., Unterberg M., Adamzik M., Koos B. (2024). Pathogens.

[cit6] van Bergenhenegouwen J., Plantinga T. S., Joosten L. A. B., Netea M. G., Folkerts G., Kraneveld A. D., Garssen J., Vos A. P. (2013). J. Leukocyte Biol..

[cit7] Kaesler S., Skabytska Y., Chen K.-M., Kempf W. E., Volz T., Köberle M., Wölbing F., Hein U., Hartung T., Kirschning C., Röcken M., Biedermann T. (2016). J. Allergy Clin. Immunol..

[cit8] Triantafilou K., Triantafilou M., Ladha S., Mackie A., Dedrick R. L., Fernandez N., Cherry R. (2001). J. Cell Sci..

[cit9] Triantafilou M., Mouratis M.-A., Lepper P. M., Haston R. M., Baldwin F., Lowes S., Ahmed M. A. E., Schumann C., Boyd O., Triantafilou K. (2012). Virulence.

[cit10] Kinsner A., Pilotto V., Deininger S., Brown G. C., Coecke S., Hartung T., Bal-Price A. (2005). J. Neurochem..

[cit11] Nilsen N. J., Deininger S., Nonstad U., Skjeldal F., Husebye H., Rodionov D., von Aulock S., Hartung T., Lien E., Bakke O., Espevik T. (2008). J. Leukocyte Biol..

[cit12] Draing C., Traub S., Deininger S., Mang P., Möller H. M., Manso M., Rossi F., Morath S., Hartung T., von Aulock S. (2008). Eur. J. Immunol..

[cit13] Levels J. H. M., Abraham P. R., van Barreveld E. P., Meijers J. C. M., van Deventer S. J. H. (2003). Infect. Immun..

[cit14] Stadelmaier A., Morath S., Hartung T., Schmidt R. R. (2003). Angew. Chem., Int. Ed..

[cit15] Figueroa-Perez I., Stadelmaier A., Deininger S., Aulock S., Hartung T., Schmidt R. R. (2006). Carbohydr. Res..

[cit16] Deininger S., Stadelmaier A., von Aulock S., Morath S., Schmidt R. R., Hartung T. (2003). J. Immunol..

[cit17] Deininger S., Figueroa-Perez I., Sigel S., Stadelmaier A., Schmidt R. R., Hartung T., von Aulock S. (2007). Clin. Vaccine Immunol..

[cit18] Morath S., Stadelmaier A., Geyer A., Schmidt R. R., Hartung T. (2002). J. Exp. Med..

[cit19] van der Es D., Hogendorf W. F. J., Overkleeft H. S., van der Marel G. A., Codée J. D. C. (2017). Chem. Soc. Rev..

[cit20] Jimenez-Dalmaroni M. J., Xiao N., Corper A. L., Verdino P., Ainge G. D., Larsen D. S., Painter G. F., Rudd P. M., Dwek R. A., Hoebe K., Beutler B., Wilson I. A. (2009). PLoS One.

[cit21] Hong S. W., Baik J. E., Kang S.-S., Yun C.-H., Seo D. G., Han S. H. (2014). Mol. Immunol..

[cit22] Dirksen A., Hackeng T. M., Dawson P. E. (2006). Angew. Chem., Int. Ed..

[cit23] Morath S., Geyer A., Hartung T. (2001). J. Exp. Med..

[cit24] Westphal O. and Jann K., in Bacterial Lipopolysaccharides: extraction with phenol-water and further applications of the procedure, Methods in Carbohydrate Chemistry, ed. L. R. Whistler, Academic Press, New York, 1965, vol. 5 pp. 83–91

[cit25] Rismondo J. and Grundling A., in The Bacterial Cell Wall: Methods and Protocols, Methods in Molecular Biology, ed. T.-T. Hung, Humana, New York, 2024, pp. 107–124

[cit26] Groves P. (2017). Polym. Chem..

[cit27] Buist G. J., Bunton C. A. (1971). J. Chem. Soc. B.

[cit28] Buist G. J., Bunton C. A., Hipperson W. C. P. (1971). J. Chem. Soc. B.

[cit29] Dirksen A., Hackeng T. M., Dawson P. E. (2006). Angew. Chem., Int. Ed..

[cit30] Price C. C., Knell M. (1942). J. Am. Chem. Soc..

[cit31] Sussich F., Cesàro A. (2000). Carbohydr. Res..

[cit32] Aalmo K. M., Painter T. J. (1981). Carbohydr. Res..

[cit33] Kaneko T., Osaka T., Inagaki M., Habe K., Okabe T., Tsuneda S. (2025). J. Virol..

[cit34] Millership C. and Grundling A., in The Bacterial Cell Wall: Methods and Protocols, Methods in Molecular Biology, ed. T.-T. Hung, Humana, New York, 2024, 95–106

[cit35] Kang S.-S., Sim J.-R., Yun C.-H., Han S. H. (2016). Arch. Pharm. Res..

[cit36] Brightbill H. D., Modlin R. L. (2000). Immunology.

[cit37] Schröder N. W. J., Morath S., Alexander C., Hamann L., Hartung T., Zähringer U., Göbel U. B., Weber J. R., Schumann R. R. (2003). J. Biol. Chem..

[cit38] Fox J. M., Letellier E., Oliphant C. J., Signoret N. (2011). Blood.

[cit39] Shiraishi T., Yokota S., Sato Y., Ito T., Fukiya S., Yamamoto S., Sato T., Yokota A. (2018). Benefic. Microbes.

[cit40] Kaesler S., Skabytska Y., Chen K.-M., Kempf W. E., Volz T., Köberle M., Wölbing F., Hein U., Hartung T., Kirschning C., Röcken M., Biedermann T. (2016). J. Allergy Clin. Immunol..

[cit41] Ofek I., Beachey E. H., Jefferson W., Campbell G. L. (1975). J. Exp. Med..

[cit42] Beachey E. H., Dale J. B., Simpson W. A., Evans J. D., Knox K. W., Ofek I., Wicken A. J. (1979). Infect. Immun..

[cit43] Alam J. M., Yamazaki M. (2011). Chem. Phys. Lipids.

[cit44] Startek J. B., Talavera K., Voets T., Alpizar Y. A. (2018). Sci. Rep..

[cit45] Schröder N. W. J., Morath S., Alexander C., Hamann L., Hartung T., Zähringer U., Göbel U. B., Weber J. R., Schumann R. R. (2003). J. Biol. Chem..

[cit46] Jiménez-Dalmaroni M. J., Radcliffe C. M., Harvey D. J., Wormald M. R., Verdino P., Ainge G. D., Larsen D. S., Painter G. F., Ulevitch R., Beutler B., Rudd P. M., Dwek R. A., Wilson I. A. (2015). Innate Immun..

[cit47] Kelley S. L., Lukk T., Nair S. K., Tapping R. I. (2013). J. Immunol..

[cit48] Kubicek-Sutherland J. Z., Hengartner A. C., Mukundan H. (2017). Trans. Mater. Res. Soc. Jpn..

[cit49] Kubicek-Sutherland J. Z., Vu D. M., Noormohamed A., Mendez H. M., Stromberg L. R., Pedersen C. A., Hengartner A. C., Klosterman K. E., Bridgewater H. A., Otieno V., Cheng Q., Anyona S. B., Ouma C., Raballah E., Perkins D. J., McMahon B. H., Mukundan H. (2019). Sci. Rep..

[cit50] Stromberg L. R., Hengartner N. W., Swingle K. L., Moxley R. A., Graves S. W., Montaño G. A., Mukundan H. (2016). PLoS One.

