# Supplementary material for: A two-step bioconjugation of S. aureus lipoteichoic acid (LTA) affords fluorescent probes that illuminate the interaction between Gram-positive glycolipids and mammalian cell membranes
Source: RSC Chem Biol. 2026 May 26;7(7):1402–8. doi: 10.1039/d6cb00113k (PMC13231905; doi:10.1039/d6cb00113k)
Supplement: CB-007-D6CB00113K-s001 [file CB-007-D6CB00113K-s001.pdf]

Supplementary Information (SI) for RSC Chemical Biology.
This journal is © The Royal Society of Chemistry 2026

Supplementary Information

A two-step bioconjugation of *S. aureus* lipoteichoic acid (LTA) affords fluorescent probes that illuminate the interaction between Gram-positive glycolipids and mammalian cell membranes

Jake Henry[1,3], Joe Nabarro[1], Harriet Chidwick[1], Georgia Conrich-Wilks[2], Emily G. Morton-West[1], Alice Crook[2], Dave Boucher[2,4], Nathalie Signoret*[3,4] and Martin A. Fascione*[1,4]

[1]Department of Chemistry, [2]Department of Biology University of York, Heslington, York, YO10 5DD, UK. [3]Experimental Medicine and Biomedicine Group, Hull York Medical School, University of York, YO10 5DD. [4]York Biomedical Research Institute, University of York, Heslington, York, YO10 5DD, UK.

Keywords: lipoteichoic acid, bioconjugation, glycolipid, toll-like receptors, LTA

Methods

LTA Oxidation and Fluorophore Conjugation

Ultrapure and biologically active LTA from *S. aureus* (Invivogen catalogue: tlrl-pslta[1]) was resuspended in 200 mM NaOAc pH 5.5 to a concentration of 2 mg/mL. Of this mixture, 1.5 mL was mixed with 1.5 mL of 10 mM $NaIO_{4(aq)}$ (LTA final concentration: 1 mg/mL, $NaIO_{4(aq)}$ final concentration: 5 mM). The reaction mixture was place on ice and wrapped in foil to protect it from light and left to mix for two hours. After two hours of stirring, the reaction was transferred evenly into two 3.5 kDa 3 mL dialysis cassettes (Thermo Scientific catalogue: 66330) and dialyzed overnight in 1 litre of 200 mM NaOAc pH 4.5 at room temperature. During the dialysis, the dialysis solvent was replaced twice. The reaction mixture (~3 mL) was aspirated from the cassettes and placed in two glass vials. 10 µL of 1 mM aniline and 60 µL of 10 mg/mL Alexa Fluor 647 (ThermoFisher catalogue: A30632) or Alexa Fluor 488 (ThermoFisher catalogue: A30629) (final Alex Fluor concentration: 380 µM, final aniline concentration: 6.5 mM) were added to their respective vials and dialyzed against 1 litre d. H_2O for two hours at room temperature using 3.5 kDa dialysis cassettes. The cassettes were finally dialyzed once more against 100 mL of d. H_2O for an additional four hours at room temperature.

NMR Analysis of Fluorescent and unmodified LTA

After purification, AF488-LTA, AF-647 LTA, and unmodified LTA were transferred to separate 15mL falcon tubes and were flash frozen in liquid nitrogen. The conical tubes were then lyophilized until all that remained was a dry powder. The powder was resuspended in 700µL of D_2O 99% and transferred to an NMR tube at concentrations of 3.8 mg/mL AF488-LTA, 2.3 mg/mL AF-647 LTA and 3.6 mg/mL unmodified LTA. The samples were analyzed on a Bruker 700MHz Neo, TXI prodigy cryoprobe H&F/C/N/D for a standard proton NMR scan (total of 256 scans each). A DOSY NMR scan was performed for each sample with the following settings:

Pulse Program	dstebpgp3s
Number of Scans	128
D20	0.15 seconds
P30	500 microseconds
Gradient	5%/95%
D1	5 seconds

Spectra were initially prepared and analyzed in Mestrenova (version 15.1.0-37919) and the final figures were prepared in Corel Draw (2025).

Calculation of polymer length in AF488- AF647-LTA and unmodified LTA, and Labelling Efficiency in AF488- AF647-LTA

Per Rismondo and Gründling 2024,[2] the Labelling efficiency was calculated by using DOSY of unmodified LTA to integrate the lipids between region of 0.75-1.5 ppm, and set value to ~59 protons. The overall number of glycerol phosphate repeats was calculated by integrating the peaks between 4.10-3.85 and divided by 5 (note: 5H per

glycerol repeat). D-ala and GlcNAc repeats were calculated similarly using integration of CH_3 peaks at ~2.1 ppm and ~1.6 ppm respectively, and divided by 3. The % substitution was calculated by dividing the number of D-Ala or D-GlcNAc substituents by number of glycerol phosphate repeats. For AlexaFluor488-LTA, the percentage labelling was calculated by integrating the lipid region to 59 protons, and then integrating smallest peak in aromatic region (1 proton) = ~75% labelling. For Alexa Fluor 647-LTA (AF488-LTA), some lipids are obscured by fluorophore peaks, so using the pre-calculated ~18 glycerol phosphate repeats in the backbone, the 4.10-3.85 ppm range was integrated and the value set to 90 (18 repeats x 5H per repeat) and then integrate the dd peak at ~6.5 ppm (assuming 2 alkene fluorophore protons overlapping[3]), this value integrates to 3.74 H, indicative of labelling with ~1.87 AlexaFlour647 fluorophores per LTA molecule (based on 18 glycerol phosphate repeats). Similarly using only the 2 x CH_3 peak for the lipid anchor at ~0.75 ppm (integration set to 6H) also calculates approximately ~1.87 Alexa Flour 647 fluorophores per LTA molecule for AF647-LTA.

TSDS-PAGE analysis of Fluorescent LTA

2x loading dye was added to AF488- or AF647-LTA (stocks at 1 mg/mL) as well as *E. coli* (O111:B4) lipopolysaccharide FITC as a ladder (1 mg/ml Sigma Aldrich F3665-1MG). Samples were heated to 60 °C for 20 minutes then left to return at room temperature for 10 minutes. Gel tank and buffers were left to sit in an icebox to equilibrate for 20 minutes and the electrophoresis was run on ice. Following an empty gel pre-run of 10 minutes at 20 mA with the upper cathode buffer, samples were loaded and run until reaching the stacking/ separating gel interface (~1 hour). The cathode buffer was then exchanged for the lower cathode buffer and the voltage was increased to 80mA for the rest of the run. The gel was then washed 3x in MilliQ water and taken for fluorescent imaging.

<u>Materials for TSDS Gel</u>

2x Loading dye (1 ml)

- Tris-Cl pH 6.8 0.125 M
- Glycerol 40%
- Sodium dodecyl sulphate (SDS) 4% w/v
- Sodium deoxycholate (DOC) 1% w/v
- Bromophenol blue 0.005% w/v
- MilliQ H_2O up to 1 ml

16% (w/v) acrylamide resolving gel (30 ml)

- Tris-Cl pH 8.45 0.75 M
- 30% (w/v) acrylamide /0.8% (w/v) bis-acrylamide 16% w/v
- SDS 0.1% w/v
- DOC 0.05% w/v
- *d.* H_2O up to 29.75 ml
- 10% w/v ammonium persulfate 0.075% w/v
- Tetramethyl ethylenediamine 5.82 x 10^{-4} µg/ml
-

6% (w/v) acrylamide stacking gel (12 ml)

- Tris-Cl pH 6.8
- 30% (w/v) acrylamide /0.8% (w/v) bis-acrylamide 6% w/v
- SDS 0.1% w/v
- DOC 0.05% w/v
- *d.* H_2O
- 10% w/v ammonium persulfate 0.075% w/v
- Tetramethyl ethylenediamine 5.82 x 10^{-4} µg/ml

Anode Buffer

- Tris-Cl pH 8.9 0.2 M
- (0.2 µm filtered before use)

Upper Cathode Buffer

- Tris 0.1 M
- Tricine 0.1 M
- SDS 0.001% w/v

Lower Cathode Buffer

- Tris 0.1 M
- Tricine 0.1 M
- SDS 0.01% w/v

The gel was imaged directly for fluorescence using an Amersham Typhoon 5 Biomolecular imager using settings reported in the table below and a composite image was created to include both colours of modified LTA processed using FIJI (ImageJ 1.54p).

**Channel**	**PMT**	**Power/V**	**Emission Filter**	**Scan Speed**	**Resolution/ µm**
AF488	Multi-alkali	305	525BP20	Normal	10
AF647	Multi-alkali	275	670BP30	Normal	10

Detection of TLR-2 Expression on HEK-mTLR2/6 and HEK-mTLR1/2 cell lines

Parental HEK-293 LacZ (HEK-293) cells and derived lines overexpression the murine TLR2/1 or TLR1/2 receptor pairs (All from Invivogen) were detached in PBS-EDTA and were labelled in suspension with 5µg/ml mouse anti-TLR2 (Clone T2.5; BioLegend 1218021) for 1h in ice-cold FACS buffer (PBS 1% FCS Azide). Cells were washed 3 time in cold FACS buffer before being stained with a secondary polyclonal goat anti-mouse Alexa 594 (1/500). Samples were run on a Cytoflex S flow cytometer (Beckman Coulter) and results analysed using the FlowJo v10 software (Becton Dickinson).

Detection of AF488 LTA Binding to HEK-293, HEK-mTLR2/6 or HEK-mTLR1/2 cells by flow cytometry

Cells detached in PBS 10 mM EDTA were incubated in suspension with 0 to 10 µg/ml of unmodified, AF-488- or AF-647-labelled LTA in PBS for 90 mins on ice as indicated in text, before performing 3 washes in ice cold FACS buffer. Samples were either analysed directly by flow cytometry, or labelled on ice using a mouse anti-LTA monoclonal antibody (clone 55, Invivogen catalogue MA1-40134) followed by an AF-647 coupled secondary anti-mouse polyclonal for detection. All samples were run on a LRSFortessa X20 (Beckton Dickinson) or a Cytoflex S (Beckman Coulter) flow cytometer recording the mean intensity fluorescence (MFI) values of cell-bound fluorescence and results were analysed using the FlowJo v10 software. Graphs plot results from experiments performed in triplicate.

Colocalization of AF488-LTA and TLR2 on human monocyte-derived macrophages (MDMs)

Primary human monocytes were differentiated for 7 days using 50 ng/mL macrophage colony-stimulating factor M-CSF into MDMs. Following differentiation, 100,000 cells were plated on glass coverslips in four well polypropylene dishes and left to adhere overnight before labelling on ice with the mouse anti-human TLR2 antibody at 1:100 final dilution and staining with secondary AF594-anti-mouse antibody (Life Technologies A11020 2 mg/mL) to a final dilution of 1:500. Coverslips were washed and incubated with 10 µg/mL AF-488 LTA for 2h before being fixed in 3% formaldehyde for 20 minutes at room temperature. Free aldehyde groups were quenched using 50 mM NH_4Cl. They were then mounted using Mowviol+DAPI and imagedon a Zeiss LSM980 upright confocal microscope.

Detection of IL-8 Production as a result of AF488-LTA Treatment

Assay performed as previously described by Fox et al. Blood 2011[3] using Peprotech's Mini TMB EDK IL-8 ELISA development kit (catalog 900-TM18).

Deacylation of LTA by Alkaline Hydrolysis

Alkaline hydrolysis was performed following the method of Cot et al. 2011.[4] 0.5 mg fluorescent-LTA was mixed with 1M sodium hydroxide at 37 °C with no further stirring following mixing. The reaction was stopped by buffer exchange into distilled water (17,000 x g, 4 °C, 3 x 5 minutes) using a 3 kDa MWCO spin concentration tube.

700 MHz ^{1}H-NMR Overlay of Unmodified LTA with DOSY filter

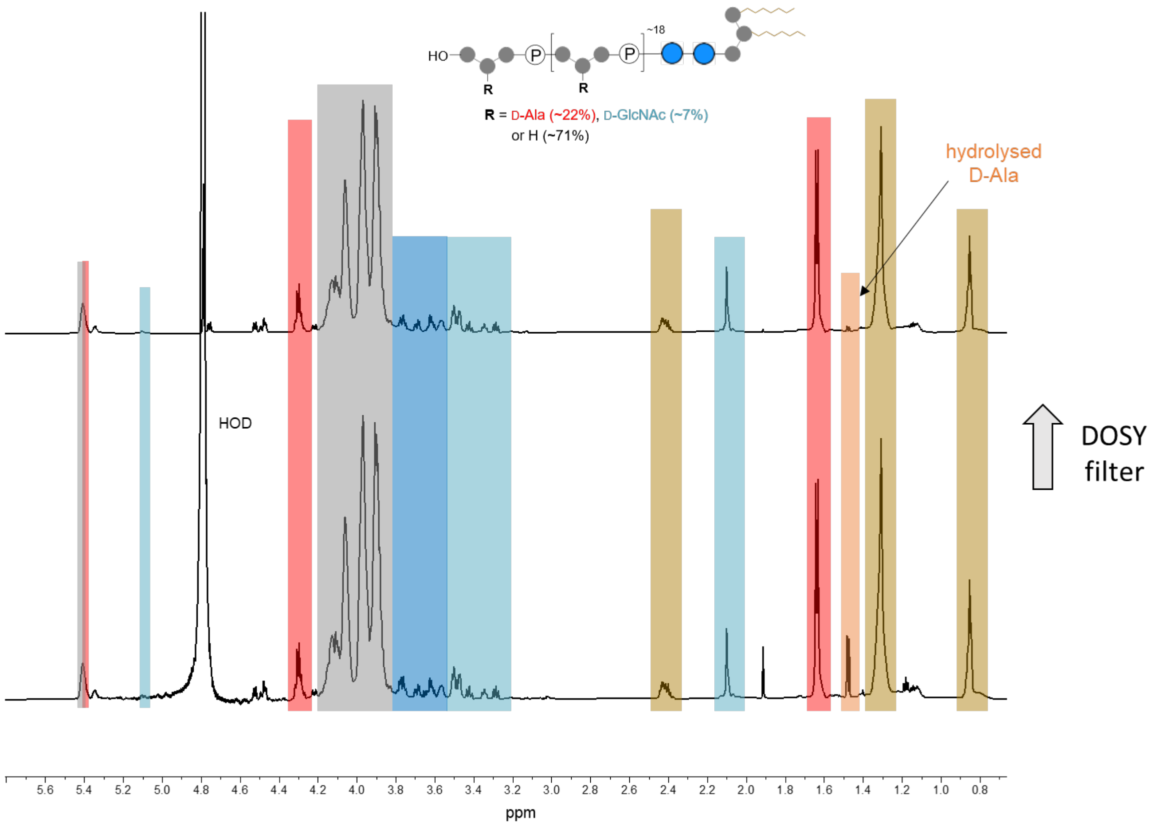

Supplemental Figure S1. **^{1}H-NMR Overlay Unmodified LTA with DOSY filter.** Unmodified-LTA without DOSY filter (bottom spectrum) and unmodified LTA with DOSY filter (top spectrum) indicating presence of hydrolysed D-Ala in commercial *S. aureus* LTA (loss of ~5% substitution from backbone).

700 MHz DOSY ¹H-NMR Overlay of AF488-LTA with Unmodified LTA

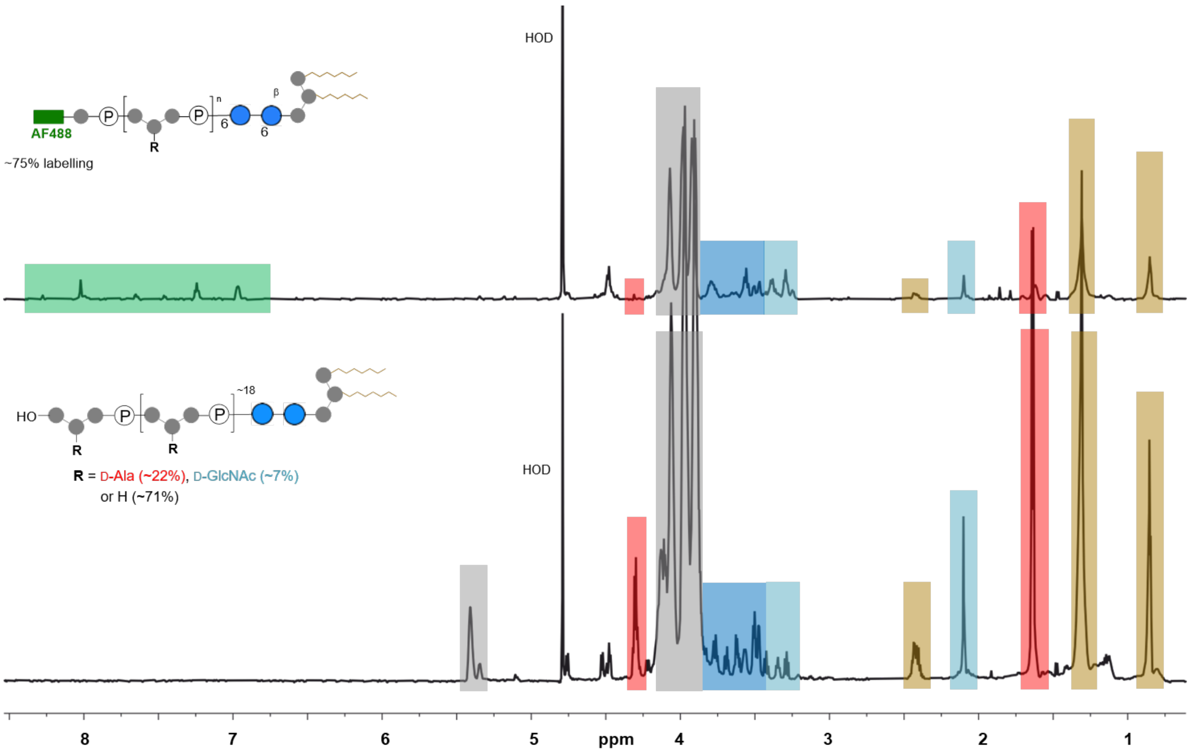

Supplemental Figure S2. **DOSY ¹H-NMR Overlay of AF488-LTA and Unmodified LTA.** AF488-LTA (top spectrum) and unmodified LTA (bottom spectrum) were resuspended in 700 µL of D_2O and analyzed via proton NMR.

700 MHz DOSY ^{1}H-NMR Overlay of AF647-LTA with Unmodified LTA

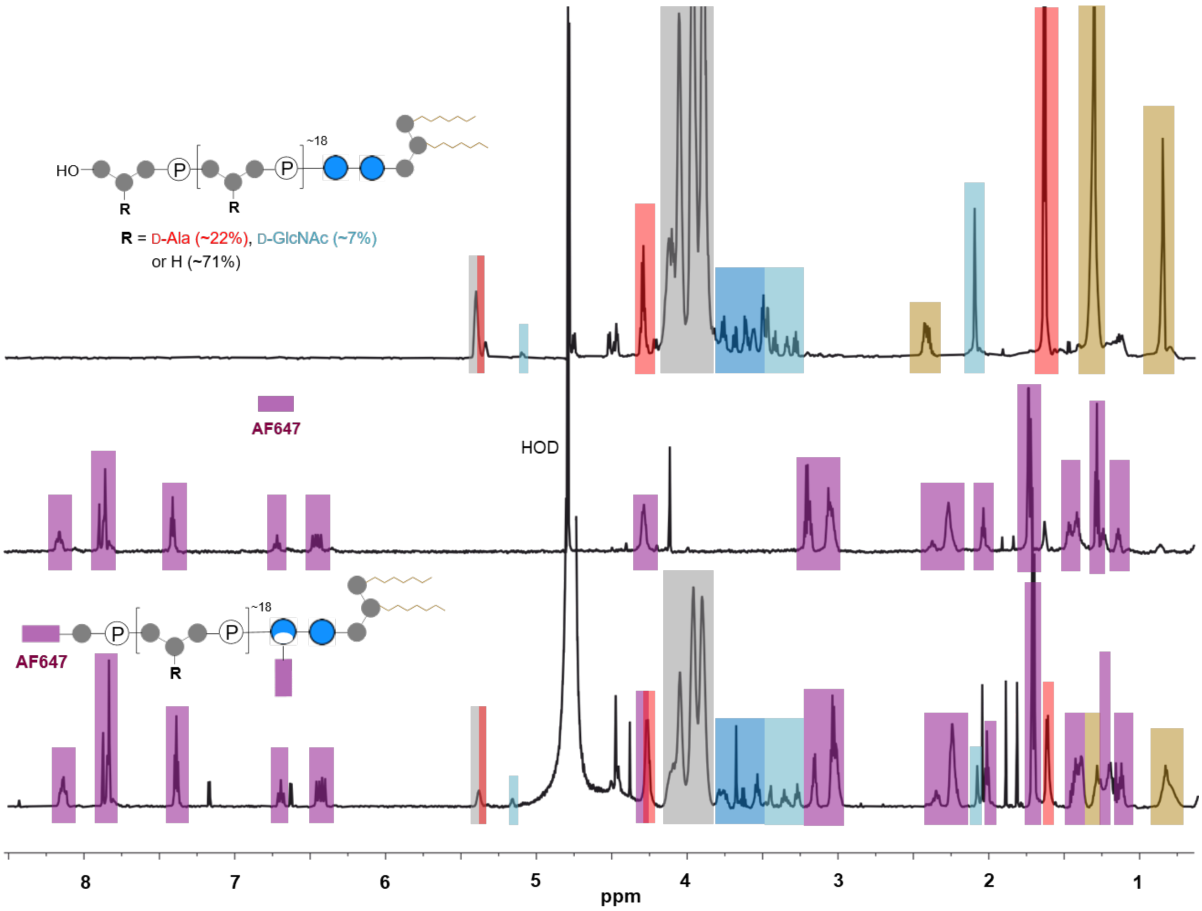

Supplemental Figure S3. **DOSY ^{1}H-NMR Overlay of AF647-LTA, AF-647 and Unmodified LTA.** Unmodified LTA (top spectrum), AlexaFluor-647 (middle spectrum), and AF647-LTA (bottom spectrum) were resuspended in 700 µL of D_2O and analyzed via proton NMR.

Comparative AF-LTA binding to HEK-293 TLR2 negative cells

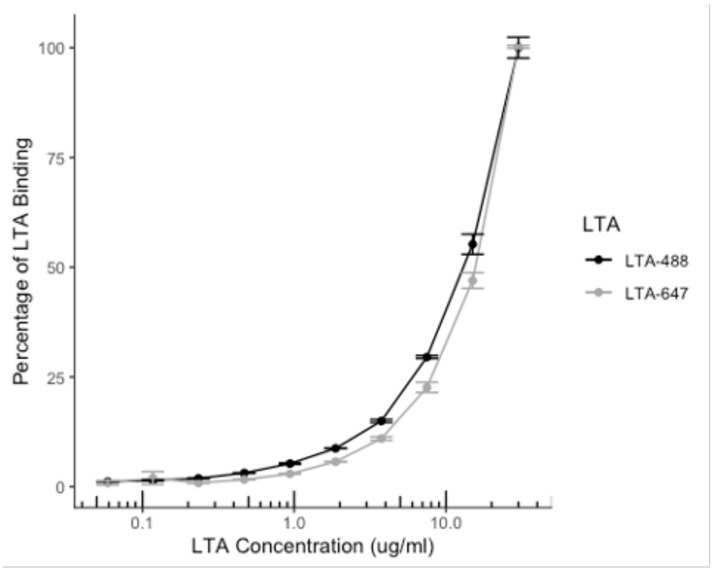

Supplemental Figure S4. **Comparative binding titration of AF488-TLA and AF647-LTA on HEK-293 control cells** showing similar non saturating concentration dependent increase in cell association after a 90 minutes treatment at 4°C, as measured by flow cytometry; results are expressed as % of maximal LTA binding for each molecule.

AF-647-LTA hydrolysis and impact on HEK-293 control cells binding

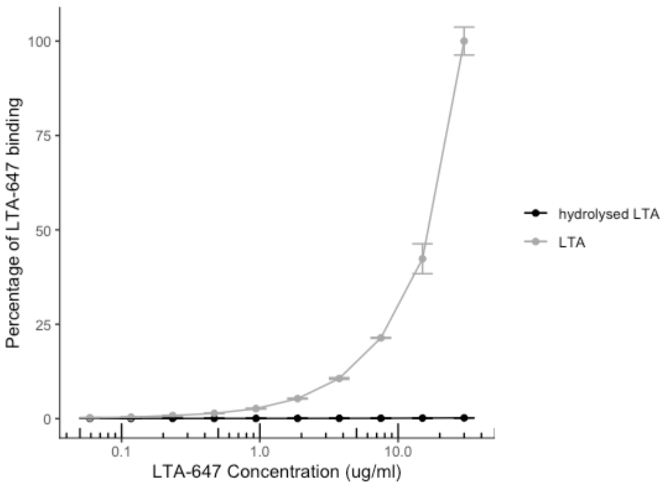

***Supplemental Figure S5*. Comparative Binding of hydrolysed and non-hydrolysed AF488-647binding on HEK-293 cells.** Cell interaction is lost when LTA is hydrolysed. This shows similar effects to figure 5F in the main manuscript where hydrolysed AF488-LTA was unable to bind to wild type HEK cells or HEK cells with TLR1/2 and TLR2/6 constructs. Results from flow cytometry analysis are expressed as % of maximal LTA binding for each molecule.

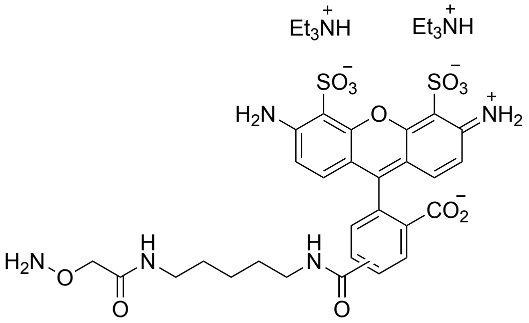

Alexa Fluor 488 hydroxylamine

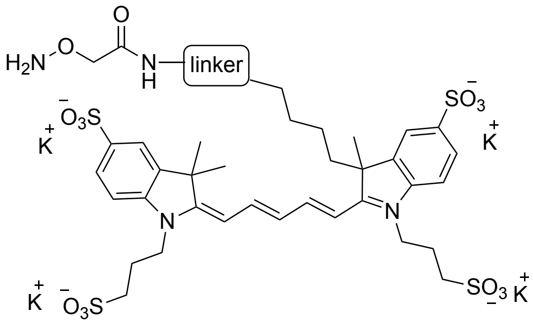

Alexa Fluor 647 hydroxylamine

Supplemental Figure S6. Structure of Alexa Fluor 488-hydroxylamine and partial structure of Alexa fluor 647 hydroxylamine (full linker structure not provided by supplier).

References

1 M. R. Kimbrell, H. Warshakoon, J. R. Cromer, S. Malladi, J. D. Hood, R. Balakrishna, T. A. Scholdberg and S. A. David, *Immunol. Lett.*, 2008, **118**, 132–141.

2 J.Rismondo, A. Grundling, in *The Bacterial Cell Wall: Methods and Protocols, Methods in Molecular Biology*, ed. Hung Ton-That, Humana, New York, 2024,107-124.

3 J. M. Fox, E. Letellier, C. J. Oliphant and N. Signoret, *Blood*, 2011, **117**, 1851–1860.

4 M. Cot, A. Ray, M. Gilleron, A. Vercellone, G. Larrouy-Maumus, E. Armau, S. Gauthier, G. Tiraby, G. Puzo and J. Nigou, *PloS One*, 2011, **6**, e26316.
